# Two cationic porphyrin isomers showing different multimeric G-quadruplex recognition specificity against monomeric G-quadruplexes

**DOI:** 10.1093/nar/gku526

**Published:** 2014-06-17

**Authors:** Xiao-Xi Huang, Li-Na Zhu, Bin Wu, Yan-Fang Huo, Na-Na Duan, De-Ming Kong

**Affiliations:** 1 State Key Laboratory of Medicinal Chemical Biology, Nankai University, Tianjin 300071, PR China; 2 Collaborative Innovation Center of Chemical Science and Engineering (Tianjin), Tianjin 300071, PR China; 3 Department of Chemistry, Tianjin University, Tianjin 300072, PR China

## Abstract

Ligands that can interact specifically with telomeric multimeric G-quadruplexes could be developed as promising anticancer drugs with few side effects related to other G-quadruplex-forming regions. In this paper, a new cationic porphyrin derivative, *m*-TMPipEOPP, was synthesized and characterized. Its multimeric G-quadruplex recognition specificity under molecular crowding conditions was compared to its isomer *p*-TMPipEOPP. The slight structural difference accounts for different multimeric G-quadruplex recognition specificity for the two isomers. *p*-TMPipEOPP can barely discriminate between multimeric and monomeric G-quadruplexes. By contrast, *m*-TMPipEOPP can bind with multimeric but not with monomeric G-quadruplexes. *p*-TMPipEOPP might bind to multimeric G-quadruplexes by two modes: sandwich-like end-stacking mode and pocket-dependent intercalative mode. Increasing the pocket size between adjacent two G-quadruplex uints is beneficial for the latter mode. *m*-TMPipEOPP might bind to multimeric G-quadruplexes by a side binding mode, which confers *m*-TMPipEOPP with higher multimeric G-quadruplex recognition specificity compared to *p*-TMPipEOPP. *m*-TMPipEOPP increases the stability of multimeric G-quadruplex under both dilute and molecular crowding conditions but its G-quadruplex-stabilizing ability is a little weaker than *p*-TMPipEOPP. These results provide important information for the design of highly specific multimeric G-quadruplex ligands. Another interesting finding is that pocket size is an important factor in determining the stability of multimeric G-quadruplexes.

## INTRODUCTION

A G-quadruplex is a unique, highly ordered nucleic acid structure formed by guanine (G)-rich deoxyribonucleic acid (DNA) or ribonucleic acid (RNA) (1–3). Gene sequences able to form G-quadruplexes exist in many essential areas of the human genome (4–6), and there is growing evidence that G-quadruplex structures are involved in some key cellular processes, including transcription, recombination and replication (4–9). As a result, G-quadruplexes are considered as novel targets for drug discovery (10). For example, as a promising anticancer target, G-quadruplex formation at telomeric regions can inhibit telomerase activity and interfere with telomere biology (7,11–13). Therefore, the development of highly specific telomeric G-quadruplex ligands as new anticancer drugs has attracted increasing attention (13–17).

Numerous studies have been conducted to investigate the interactions between candidate G-quadruplex ligands and G-quadruplexes formed by telomeric DNAs (18–23). A controversial issue in these studies, however, is the selection of target G-quadruplexes. That is, most of these studies have focused on short telomeric DNA sequences of 20−30 bases (3,24–26), which can form only monomeric G-quadruplexes containing a single G-quadruplex unit. In fact, the human telomeric sequence consists of thousands of TTAGGG repeats terminated with a 3′-end single-stranded overhang of ∼200 nt. Some studies have demonstrated such a G-rich single-stranded overhang can fold into G-quadruplexes composed of several G-quadruplex units (27–32). We have reason to believe ligands targeting telomeric multimeric G-quadruplexes are the most promising candidates for anticancer drugs (33–35).

As well as the telomeric sequence, there are >370 000 putative G-quadruplex-forming sequences in the human genome (36,37). In these sequences, however, only the telomeric sequence has the ability to form multimeric G-quadruplexes. That is to say, the ligands that can interact specifically with telomeric multimeric G-quadruplexes could be developed as promising anticancer drugs with few side effects related to other potential G-quadruplex-forming regions (35,38).

As well as the selection of a G-rich sequence, reaction conditions should be considered in G-quadruplex/ligand interaction studies. Current studies are commonly done under dilute conditions. The interior of cells, however, is a crowded environment containing many biological molecules (39). Molecular crowding environment might induce change of the conformation and stability of G-quadruplexes and, thus, influence their binding behavior with ligands (40–45). It has been demonstrated that monomeric G-quadruplex-stabilizing ligands that work very well under dilute conditions showed reduced or even negligible monomeric G-quadruplex-stabilizing abilities under molecular crowding conditions (46). Although no study has compared the stabilizing ability of ligands to multimeric G-quadruplexes under different conditions, it has been demonstrated that multimeric G-quadruplexes formed by long telomeric sequences have different G-quadruplex structures under dilute compared to molecular crowding conditions (31). That is to say, it is highly likely some ligands display a different ability to bind multimeric G-quadruplexes under different conditions. Overall, investigating the interactions between ligands and multimeric G-quadruplexes under molecular crowding conditions is a direct and effective route to screen new kinds of anticancer drugs that specifically target telomeric regions.

**Scheme 1.**
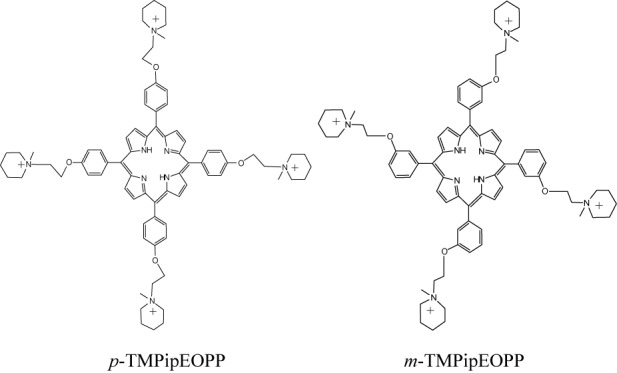
The chemical structures of *p*-TMPipEOPP and *m*-TMPipEOPP.

Recently, we designed a cationic porphyrin derivative with four large side arm substituents, 5,10,15,20-tetra-{4-[2-(1-methyl-1-piperidinyl)ethoxy] phenyl} porphyrin (*p*-TMPipEOPP; Scheme 1), and the possibility of this derivative acting as a ligand of multimeric G-quadruplexes was investigated under molecular crowding conditions (47). The results showed this porphyrin derivative could promote the formation of multimeric G-quadruplexes and stabilize them. *p*-TMPipEOPP lacks the ability to distinguish between multimeric and monomeric G-quadruplexes, however, implying the telomeric region is not the sole target of this ligand. In this study, we synthesized another cationic porphyrin derivative, 5,10,15,20-tetra-{3-[2-(1-methyl-1-piperidinyl)ethoxy] phenyl} porphyrin (*m*-TMPipEOPP; Scheme 1). *m*-TMPipEOPP and *p*-TMPipEOPP are isomers. The slight change of structure accounts for *m*-TMPipEOPP with a higher level of selectivity for multimeric G-quadruplexes compared to *p*-TMPipEOPP. Different binding modes to multimeric G-quadruplexes formed by several telomeric sequences with different lengths and their mutants were proposed for these two porphyrin derivatives on the basis of comparison of their binding behavior.

**Figure 1.**
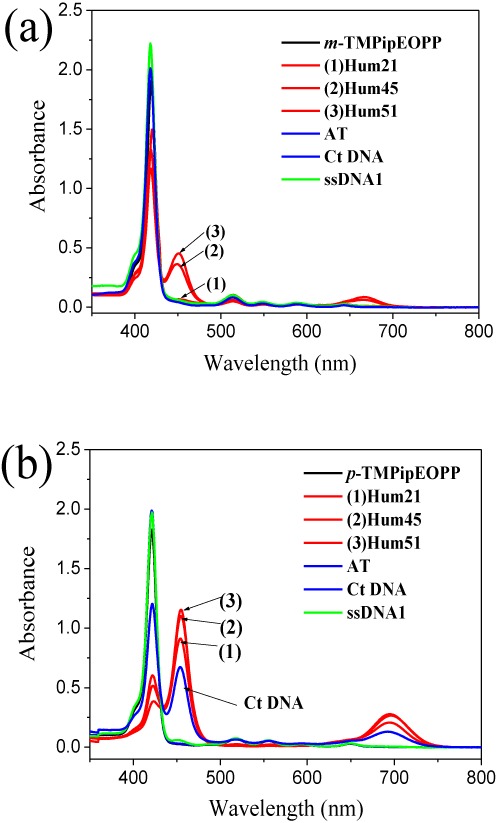
UV-vis absorption spectra of *m*-TMPipEOPP (**a**) and *p*-TMPipEOPP (**b**) in absence or presence of different DNAs. Free porphyrin (black line); G-quadruplex (red line); duplex DNA (blue line); single-stranded DNA (green line). [Porphyrin] = 2.5 μM; [multimeric quadruplex] = 10 μM; [monomeric quadruplex] = [duplex DNA] = [single-stranded DNA] = 20 μM; [Ct DNA] = 1200 μM (base concentration). The effects of other DNAs on the absorption spectrum of *m*-TMPipEOPP were shown in Supplementary Figure S4.

**Figure 2.**
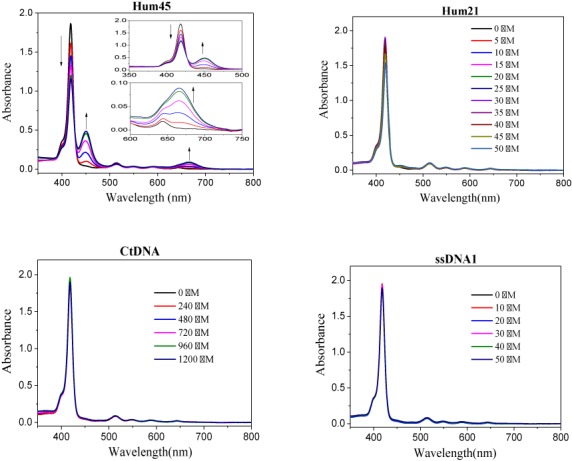
DNA concentration-dependent absorption spectrum changes of *m*-TMPipEOPP in the presence of multimeric G-quadurplex Hum45, monomeric G-quadruplex Hum21, duplex Ct DNA or single-stranded ssDNA1. The concentrations of Hum45 are (arrow direction): 0, 2.5, 5, 10, 15 and 20 μM. The concentrations of other DNAs are labeled in the figures. Except the concentration of Ct DNA is represented as base concentration, other DNAs are all represented as single-stranded concentration. DNA concentration-dependent *m*-TMPipEOPP spectrum changes in the presence of other DNAs can be found in Supplementary Figures S5–S9.

**Figure 3.**
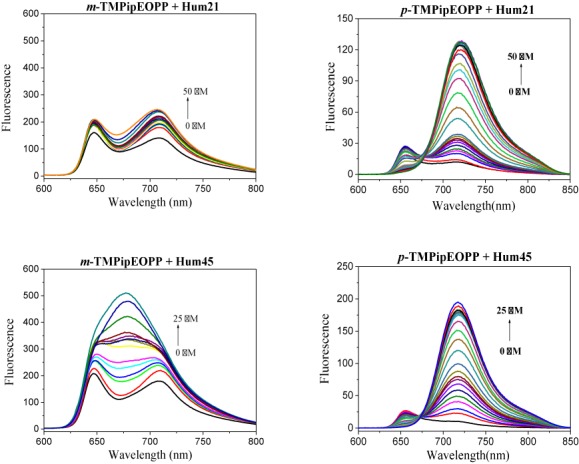
Fluorescence spectra of *m*-TMPipEOPP (left) and *p*-TMPipEOPP (right) in the presence of different concentrations of monomeric G-quadruplex Hum21 or multimeric G-quadruplex Hum45. The effects of other multimeric G-qudruplexes (Hum51, Hum 57, Hum63, Hum69 and their mutants) on the fluorescence spectra of these two porphyrin isomers are shown in Supplementary Figure S10.

## MATERIALS AND METHODS

### Materials and reagents

The oligonucleotides listed in Table 1 and calf thymus DNA (Ct DNA) were purchased from Sangon Biotech. Co. Ltd. (Shanghai, China). The concentrations of the oligonucleotides were represented as single-stranded concentrations. Single-stranded concentrations were determined by measuring the absorbance at 260 nm. Molar extinction coefficient was determined using a nearest neighbor approximation (http://www.idtdna.com/analyzer/Applications/OligoAnalyzer), and the calculated molar extinction coefficients of these oligonucleotides were listed in Table 1. The concentration of Ct DNA was represented as the base concentration, which was determined by the absorbance at 260 nm using the molar absorption coefficient of 6600 M^−1^ cm^−1^. The Ct DNA solution gave a ratio of ultraviolet (UV) absorbance at 260 and 280 nm of 1.83:1, which indicates that the DNA was sufficiently free of protein. Na_2_EDTA (disodium ethylenediamine tetraacetic acid), Tris (tris(hydroxymethyl)aminomethane), polyethylene glycol 200 (PEG 200), CH_3_OH, N,N-dimethylformamide (DMF) and CH_2_Cl_2_ were obtained from Sigma. 1-(2-Chloroethyl)piperidine hydrochloride was bought from Huai'an City East Chemical Factory (Jiangsu China). DMF was distilled over CaH_2_ before use. CH_2_Cl_2_ was distilled from CaH_2_ and stored over molecular sieves. Other chemical reagents were of analytical grade and used without further purification. Deionized and sterilized water (resistance > 18 MΩ/cm) was used throughout the experiments. *p*-TMPipEOPP was synthesized according to the method reported by us (48). The details of synthesis and characterization of *m*-TMPipEOPP are available in Supporting Information (Supplementary Figures S1–S3).

**Table 1.** G-rich oligonucleotides used in this work

DNA	Sequence (from 5′ to 3′)^a^	Extinction coefficient (L mol^−1^ cm^−1^)	Structure
Hum21	GGG(TTAGGG)_3_	215 000	G-quadruplex (monomeric)
Hum45	GGG(TTAGGG)_7_	459 400	G-quadruplex (multimeric)
Hum51	GGG(TTAGGG)_8_	520 500	G-quadruplex (multimeric)
Hum51-M1	GGG(TTAGGG)_7_**TGTGAG**	520 300	G-quadruplex (multimeric)
Hum51-M2	GGG(TTAGGG)_3_**TTGAGTGTA**GGG(TTAGGG)_3_	520 300	G-quadruplex (multimeric)
Hum57	GGG(TTAGGG)_9_	581 600	G-quadruplex (multimeric)
Hum57-M1	GGG(TTAGGG)_7_**TGTGAGTGTGAG**	581 200	G-quadruplex (multimeric)
Hum57-M2	GGG(TTAGGG)_3_**TTGAGTGTA**GGG(TTAGGG)_3_**TGTGAG**	581 200	G-quadruplex (multimeric)
Hum57-M3	GGG(TTAGGG)_3_**TTGAGTGTGAGTGTA**GGG(TTAGGG)_3_	581 200	G-quadruplex (multimeric)
Hum63	GGG(TTAGGG)_10_	642 700	G-quadruplex (multimeric)
Hum63-M1	GGG(TTAGGG)_7_**TGTGAGTGTGAGTGTGAG**	642 100	G-quadruplex (multimeric)
Hum63-M2	GGG(TTAGGG)_3_**TTGAGTGTA**GGG(TTAGGG)_3_**TGTGAGTGTGAG**	642 100	G-quadruplex (multimeric)
Hum63-M3	GGG(TTAGGG)_3_**TTGAGTGTGAGTGTA**GGG(TTAGGG)_3_**TGTGAG**	642 100	G-quadruplex (multimeric)
Hum69	GGG(TTAGGG)_11_	703 800	G-quadruplex (multimeric)
C-MYC	TGAGGGTGGGAGGGTGGGAA	209 700	G-quadruplex (monomeric)
KRAS	AGGGCGGTGGGAAGAGGGAAGAGGGGGAGG	322 200	G-quadruplex (monomeric)
M3Q	GAGGGAGGGAGGGAGAGGGA	222 500	G-quadruplex (monomeric)
Oxy28	GGGGTTTTGGGGTTTTGGGGTTTGGGG	253 900	G-quadruplex (monomeric)
AT	(AT)_6_	133 300	Duplex
GC	(GC)_6_	101 100	Duplex
LD	GCGCAATTGCGC	108 700	Duplex
ssDNA1	GAGCTCTCGAAAGAGCTCCGATTA	235 800	Single-stranded
ssDNA2	TAGAGCACACCTGTCCGTG	179 500	Single-stranded

^a^The underlined bold letters identify rearranged sequences that cannot be participated in the formation of G-quadruplex units.

### UV-vis absorption spectroscopy

UV-vis absorption spectra were measured on a Cary 60 UV-vis spectrophotometer (Agilent Technologies) with 1 cm-path-length micro quartz cell (40 μl, Starna Brand, UK). Solutions containing individual DNAs with designated concentration, 10 mM Tris-HCl buffer (pH 7.4), 150 mM KCl, 1 mM Na_2_EDTA and 400 ml/l PEG 200 were prepared. Each solution was heated to 95°C for 5 min to remove any aggregates, then cooled rapidly to 25°C and was allowed to incubate at 25°C for 30 min. After overnight incubation at 4°C, 3 μM of *m*-TMPipEOPP or *p*-TMPipEOPP was added and the absorption spectra in the range of 350–800 nm were recorded.

Absorption titration experiments were carried out by varying the DNA concentration but maintaining the *m*-TMPipEOPP concentration constant at 3 μM. The sample solutions were prepared as above, and the absorption spectra in the range of 350–800 nm were recorded.

Scatchard analysis was conducted as previously reported (49–51). Briefly, the change in the absorbance difference between 452 and 418 nm as a function of G-quadruplex concentration was used to construct Scatchard plots [Equation (1)]:

(1) \begin{equation*}
\frac{r}{{{C}_{\rm f} }} = n{K}_{\rm a} - {K}_{\rm a} r
\end{equation*}

Here *r* is the number of moles of bound *m*-TMPipEOPP per mole of G-quadruplex; *n* is the number of *m*-TMPipEOPP-binding sites on the G-quadruplex; *K*_a_ is the binding constant; and *C*_f_ is the free *m*-TMPipEOPP in the *m*-TMPipEOPP/G-quadruplex mixture (51).

Job plot analysis was performed by systematic variation of the molar fraction of *m*-TMPipEOPP (or *p*-TMPipEOPP) and individual G-quadruplexes while keeping a constant total concentration of 10 μM for *m*-TMPipEOPP and G-quadruplex (a constant total concentration of 5 μM for *p*-TMPipEOPP and G-quadruplex). The mixtures of *m*-TMPipEOPP (or *p*-TMPipEOPP) and G-quadruplexes were prepared as above, and the absorption signals at designated wavelengths were recorded.

### Fluorescence spectroscopy

Fluorescence spectra were measured on a SHIMADZU RF-5301PC spectrofluorimeter with 1 cm-path-length micro quartz cell (40 μl, Starna Brand, UK). Solutions containing 10 μM individual oligonucleotides, 10 mM Tris-HCl buffer (pH 7.4), 150 mM KCl, 1 mM Na_2_EDTA and 400 ml/l PEG 200 were prepared. Each solution was heated to 95°C for 5 min to remove any aggregates, then cooled rapidly to 25°C and was allowed to incubate at 25°C for 30 min. After overnight incubation at 4°C, 5 μM of *m*-TMPipEOPP or *p*-TMPipEOPP was added. Fixing the excitation wavelength at 455 nm, emission spectra in the range of 600–850 nm were collected at room temperature. When the fluorescence spectrum of *p*-TMPipEOPP was recorded, the excitation slit and emission slit were both set at 5 nm. When the fluorescence spectrum of *m*-TMPipEOPP was recorded, the excitation slit and emission slit were both set at 10 nm (the same below).

Fluorescence titration experiments were carried out by fixing the *m*-TMPipEOPP (or *p*-TMPipEOPP) concentration at 5 μM but varying the DNA concentration. The sample solutions were prepared as above, and the fluorescence spectra in the range of 600–850 nm were recorded when excited at 455 nm.

### Melting temperature (*T*_1/2_) detection of G-quadruplexes

Melting temperature (*T*_1/2_) detection of G-quadruplexes was carried out on a Cary-60 UV-vis spectrophotometer equipped with a single cell Peltier temperature control accessory. The G-quadruplex (5 μM) solution were prepared in 10 mM Tris-HCl buffer (pH 7.4) containing 50 mM KCl, 1 mM Na_2_EDTA and 0 or 400 ml/l PEG 200. The solution was heated to 95°C for 5 min, then cooled rapidly to 25°C and was allowed to incubate at 25°C for 30 min. After overnight incubation at 4°C, 0 or 5 μM *m*-TMPipEOPP was added. Then, the absorption signal at 295 nm (400 nm as control wavelength) was recorded at about 20°C. When the absorption signal became constant, the temperature was increased in steps of 1°C and the absorption signal was recorded at each temperature until the signal did not decrease any more. At each temperature, the mixture was left to equilibrate for 1 min before absorption signal was recorded.

### Circular dichroism spectroscopy

A 3 ml reaction mixture was prepared in 10 mM Tris-HCl buffer (pH 7.4) containing 1 μM individual DNA oligonculeotides, 150 mM KCl, 1 mM Na_2_EDTA, 0 or 400 ml/l PEG 200. The mixture was heated at 95°C for 5 min, cooled slowly to 25°C and then incubated at 4°C overnight. Circular dichroism (CD) spectrum of the mixture was recorded between 220 and 320 nm in 1 cm path length cuvettes on a Jasco J-715 spectropolarimeter. Spectra were averaged from three scans, which were recorded at 100 nm/min with a response time of 1 s and a bandwidth of 1.0 nm.

## RESULTS AND DISCUSSION

### Multimeric G-quadruplex recognition specificity of the porphyrin isomers against duplex, single-stranded DNAs and monomeric G-quadruplexes

Genomic DNA usually exists as a canonical double-helix structure (duplex structure). A primary requirement for ideal ligands targeting a telomeric region is that they must have a high level of G-quadruplex-binding selectivity for other DNA structures, including duplex and single-stranded DNAs. Earlier, we showed *p*-TMPipEOPP could discriminate G-quadruplexes from duplex and single-stranded DNAs with a high level of specificity (47,48). To investigate the feasibility of using *m*-TMPipEOPP as a specific G-quadruplex ligand under molecular crowding conditions, binding interactions between *m*-TMPipEOPP and both monomeric and multimeric G-quadruplexes, duplex or single-stranded DNAs were investigated by following the effects of these DNAs on the UV-vis absorption spectrum of *m*-TMPipEOPP using polyethylene glycol 200 (PEG 200) as a molecular crowding agent, and the result was compared to *p*-TMPipEOPP (Figure 1 and Supplementary Figure S4). Both free *p*-TMPipEOPP and free *m*-TMPipEOPP showed a strong Soret band centered at a wavelength of ∼418 nm in their UV-vis absorption spectra. The presence of duplex (AT, GC, LD and Ct DNA, Table 1) or single-stranded DNAs (ssDNA1 and ssDNA2) had nearly no effect on the absorption spectrum of *m*-TMPipEOPP. In the presence of multimeric G-quadruplexes formed by long human telomeric DNAs with different lengths (Hum45, Hum51, Hum57, Hum63 and Hum69), however, marked hypochromicity was observed for the Soret peak at 418 nm accompanied by the appearance of a new band centered at a wavelength of ∼452 nm. Simultaneously, a weak absorption peak emerged at ∼666 nm. These results suggest *m*-TMPipEOPP can interact with multimeric G-quadruplexes but not duplex or single-stranded DNAs, implying it might discriminate specifically multimeric G-quadruplexes from duplex and single-stranded DNAs.

Lacking specificity against duplex DNAs, especially long-stranded duplex DNAs, is a drawback faced by most reported small molecular G-quadruplex ligands (21,22,52). It is noteworthy that *m*-TMPipEOPP but not *p*-TMPipEOPP displays high multimeric G-quadruplex selectivity against long-stranded duplex DNA–Ct DNA. *p*-TMPipEOPP can specifically discriminate G-quadruplexes from short-stranded duplex DNAs (e.g. AT, GC and LD), but its selectivity for Ct DNA is not satisfactory (Figure 1). The presence of Ct DNA could cause significant changes in the UV-vis absorption spectrum of *p*-TMPipEOPP, which might impair the use of *p*-TMPipEOPP as a specific G-quadruplex ligand. On the contrary, *m*-TMPipEOPP has higher G-quadruplex recognition specificity for long duplex DNAs compared to *p*-TMPipEOPP, which makes *m*-TMPipEOPP a more suitable candidate for anticancer drugs targeting G-quadruplexes.

Another important feature of *m*-TMPipEOPP is that this porphyrin derivative has a higher level of recognition specificity for multimeric compared to monomeric G-quadruplexes. As mentioned above, screening ligands that interact with multimeric rather than monomeric G-quadruplexes can help to develop anticancer drugs specifically targeting telomeric sequences. *p*-TMPipEOPP showed a high level of G-quadruplex recognition specificity against short duplex and single-stranded DNAs but it had almost no selectivity to distinguish between monomeric and multimeric G-quadruplexes. As shown in Figure 1, both monomeric (Hum21) and multimeric (Hum45 and Hum51) G-quadruplexes could lead to significant hypochromicity of the *p*-TMPipEOPP Soret band at 421 nm, accompanied by the appearance of new bands at 454 and 695 nm. By contrast, the presence of monomeric G-quadruplex (Hum21) had nearly no effect on the *m*-TMPipEOPP absorption spectrum. Only multimeric G-quadruplexes could cause marked absorption spectrum changes: emergence of the peaks at wavelengths of 452 and 666 nm suggests multimeric but not monomeric G-quadruplexes can bind to *m*-TMPipEOPP.

To further demonstrate the multimeric G-quadruplex recognition specificity of *m*-TMPipEOPP, the DNA concentration-dependent absorption spectrum change of this porphyrin dye was investigated in the presence of different DNAs (Figure 2 and Supplementary Figures S5–S9). With the addition of DNAs without multimeric G-quadruplex-forming potential, the absorption spectrum of *m*-TMPipEOPP had almost no change, even when the DNA concentrations reached very high levels. As for the DNAs that could fold into multimeric G-quadruplexes, however, DNA concentration-dependent absorption spectrum changes (continuous decrease of the absorption signal at a wavelength of 418 nm and continuous increase of the signals at wavelengths of 452 and 666 nm) could be observed clearly. A DNA concentration-independent absorption spectrum change suggested no interaction occurred between *m*-TMPipEOPP and the DNAs without multimeric G-quadruplex-forming ability. However, DNA concentration-dependent absorption spectrum changes implied *m*-TMPipEOPP could interact with DNAs that could fold into multimeric G-quadruplexes.

To demonstrate the generality of the multimeric G-quadruplex recognition specificity of *m*-TMPipEOPP against monomeric ones, the effects of another four monomeric G-quadruplexes (M3Q, Oxy28, C-MYC and KRAS) formed by different G-rich sequences on the absorption spectrum of *m*-TMPipEOPP were investigated (Supplementary Figure S8). None of the tested monomeric G-quadruplexes could cause a marked absorption spectrum change of *m*-TMPipEOPP, even when the concentration ratio of monomeric G-quadruplex to *m*-TMPipEOPP reached a very high level (10:1). These results confirm the possibility of developing *m*-TMPipEOPP as a specific multimeric G-quadruplex recognition ligand.

### Different effects of monomeric and multimeric G-quadruplexes on the fluorescence spectra of the two porphyrin isomers

The better multimeric G-quadruplex recognition ability of *m*-TMPipEOPP compared to *p*-TMPipEOPP can also be reflected by the different effects of monomeric and multimeric G-quadruplexes on the fluorescence spectra of these two porphyrin derivatives. As shown in Figure 3 and Supplementary Figure S10, free *p*-TMPipEOPP showed two fluorescence peaks centered at wavelengths of 656 and 717 nm when excited at 455 nm. Similar fluorescence spectrum changes were caused by monomeric and multimeric G-quadruplexes; i.e. with the addition of monomeric or multimeric G-quadruplexes, the fluorescence signal at wavelength of 656 nm decreased continuously and finally almost disappeared. At the same time, the fluorescence signal at wavelength of 717 nm increased greatly, accompanied by a red shift from 717 to 719 nm. These results suggested the chemical environments provided by monomeric and multimeric G-quadruplexes for *p*-TMPipEOPP might be similar. *m*-TMPipEOPP also displayed two fluorescence peaks, which were centered at wavelengths 647 and 709 nm, respectively. Completely different fluorescence spectrum changes were caused by the addition of monomeric and multimeric G-quadruplexes. With the addition of monomeric G-quadruplex (Hum21), the fluorescence signals of the two peaks increased slightly, but the fluorescence spectral shape had nearly no change. That is, two fluorescence peaks were still observed. By contrast, with the addition of multimeric G-quadruplex, for example Hum45, although the fluorescence signals of the two peaks increased, the fluorescence signal of the valley between two peaks had the greatest fluorescence enhancement. When the concentration of DNA was high enough, the original two peaks disappeared and a new peak emerged between them. These results imply monomeric G-quadruplexes can bind to *p*-TMPipEOPP but have nearly no interaction with *m*-TMPipEOPP. Multimeric G-quadruplexes could bind both *p*-TMPipEOPP and *m*-TMPipEOPP, but they might provide different environments for these two porphyrin isomers. According to the effects of G-quadruplexes on the fluorescence spectrum of *m*-TMPipEOPP, around 900-fold multimeric G-quadruplex selectivity against monomeric ones was estimated for *m*-TMPipEOPP (Supplementary Figure S11).

### Binding modes between the two porphyrin isomers and G-quadruplexes

**Scheme 2.**
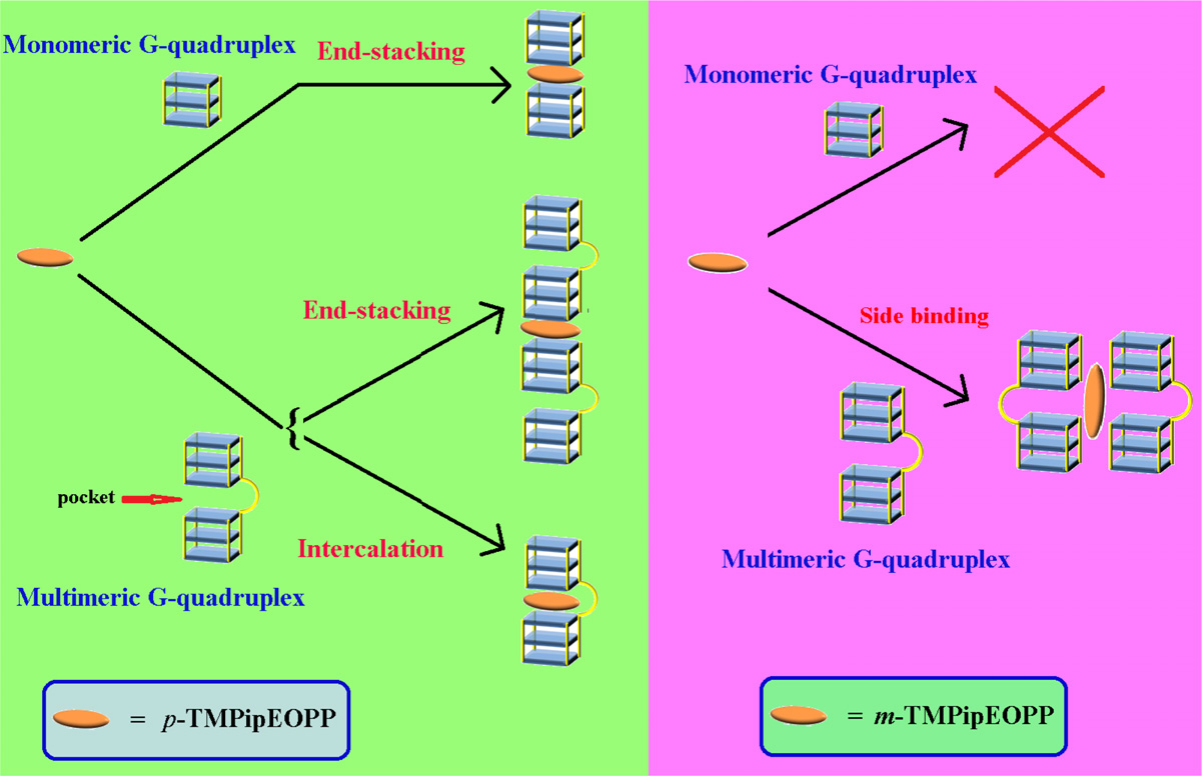
Proposed binding modes between individual two porphyrin isomers and G-quadruplexes.

The results of the experiments described above suggested *m*-TMPipEOPP has better multimeric G-quadruplex recognition specificity than *p*-TMPipEOPP against monomeric G-quadruplexes and long double-stranded DNAs. To further investigate this, the binding stoichiometry of these two porphyrin isomers to monomeric or multimeric G-quadruplexes was detected using continuous variation analysis (Job plot) (Supplementary Figures S12–S39). First, the binding stoichiometry of *p*-TMPipEOPP to monomeric (Hum21) or multimeric G-quadruplexes (Hum45, Hum51, Hum57, Hum63 and Hum69) was determined. Considering substantial changes of absorbance and fluorescence recorded for *p*-TMPipEOPP were caused by G-quadruplexes, the absorption signals at three wavelengths (421, 454 and 695 nm) and the fluorescence signal at wavelength 719 nm were used to construct Job plots, which gave similar results for these four signals. A binding stoichiometry of 1:2 was observed for the monomeric G-quadruplex Hum21; i.e. a *p*-TMPipEOPP molecule could bind two Hum21 G-quadruplexes, suggesting a sandwich-like Hum21/*p*-TMPipEOPP/Hum21 end-stacking mode might be adopted (Scheme 2). This result is consistent with our previous reports (47). As for the binding interactions between *p*-TMPipEOPP and multimeric G-quadruplexes formed by telomeric sequences of different lengths (Hum45, Hum51, Hum57 and Hum63), DNA sequence length-dependent binding stoichiometries were obtained. With increased DNA sequence length, the binding stoichiometry increased from 1:1.6 for Hum45 to 1:1 for Hum63 (Table 2). These four DNA oligonucleotides are all cut from the human telomeric sequence, but have different numbers of GGG repeats (G-tracts). Because the numbers of G-tract were all <12, they could form multimeric G-quadruplexes with two G-quadruplex units, but the multimeric G-quadruplexes formed might not be identical. Hum45 has eight G-tracts. In the multimeric G-quadruplex formed by it, the two G-quadruplex units are closely linked by a short linking TTA loop, which means the size of the pocket between adjacent two G-quadruplex units is small. Hum51 has nine tracts and two kinds of possible multimeric G-quadruplexes might be formed by it. One is two G-quadruplex units which are linked closely by nucleotides TTA and the other is two G-quadruplex units connected by a long TTAGGGTTA linker. That is, two multimeric G-quadruplexes with different sizes of pocket might be formed by Hum51. Telomeric oligonucleotides Hum57 and Hum63 have 10 and 11 G-tracts, respectively. Three kinds of multimeric G-quadruplexes might be folded by Hum57, and the maximum pocket is formed by two G-quadruplex units and a TTAGGGTTAGGGTTA linking loop. Hum63 might fold into four multimeric G-quadruplex structures.

**Table 2.** Binding stoichiometries for the interactions between G-quadruplexes and *m*-TMPipEOPP or *p*-TMPipEOPP

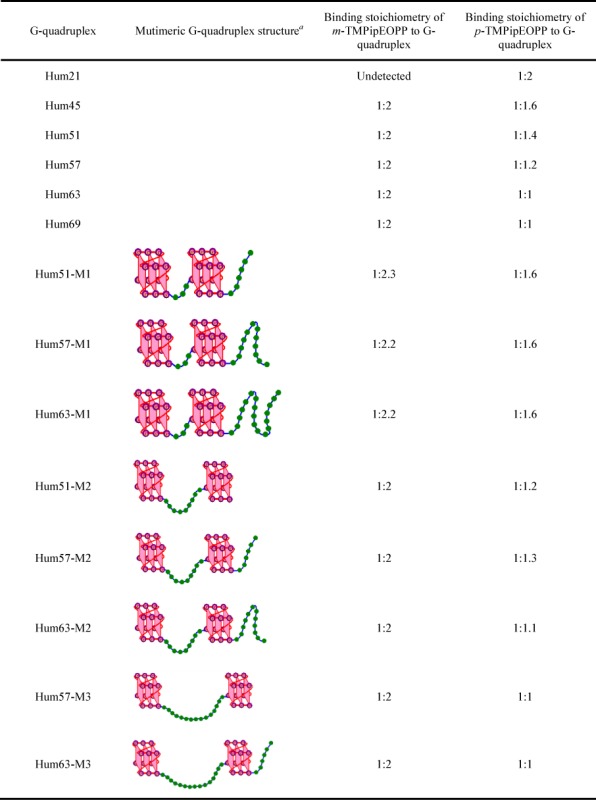			

^a^The green beads represent the nucleotide bases in the linking loop.

Although multimeric G-quadruplexes with different pocket sizes might be formed, the result of CD spectroscopy indicated the G-quadruplex units of these multimeric G-quadruplexes have strand orientation identical with the monomeric G-quadruplex formed by Hum21. That is, hybrid G-quadruplexes, which have both parallel and antiparallel strand orientations, were formed under dilute conditions (Supplementary Figure S40) and converted to parallel G-quadruplexes under molecular crowding conditions (Supplementary Figure S41). Excluding the potential effect of strand orientation on the binding interactions between *p*-TMPipEOPP and multimeric G-quadruplexes formed by telomeric sequences of different lengths, sequence length-dependent binding stoichiometry mentioned above might be interpreted by the difference in pocket size. Similar UV-vis absorption and fluorescence spectral changes of *p*-TMPipEOPP caused by monomeric and multimeric G-quadruplexes suggested they might provide similar chemical environments for *p*-TMPipEOPP (Figures 1 and 3). Thus, two binding modes can be supposed between *p*-TMPipEOPP and these multimeric G-quadruplexes (Scheme 2). One is sandwich-like end-stacking with a *p*-TMPipEOPP/G-quadruplex ratio of 1:2, the other is *p*-TMPipEOPP intercalated into the pocket between two G-quadruplex units with a binding stoichiometry of 1:1. Job plot analysis gave a *p*-TMPipEOPP/Hum45 stoichiometry of 1:1.6, suggesting two binding modes might coexist between them. With the increase of telomeric G-rich sequence length, the binding stoichiometry between *p*-TMPipEOPP and multimeric G-quadruplex increased, thus suggesting the proportion of end-stacking mode decreased, accompanied by an increased intercalative mode. As for Hum63, only the intercalative mode remained. These results suggested the binding mode might be relative to the size of the pocket between two G-quadruplex units. With the increase of pocket size, *p*-TMPipEOPP adopts the intercalative binding mode preferentially. This might be interpreted as a spatial effect; i.e. a large pocket might provide enough space for the intercalation of *p*-TMPipEOPP.

To provide evidence for the effect of pocket size, several mutants of the telomeric sequences described above were designed to form multimeric G-quadruplexes with definite pocket sizes (Table 2). Taking Hum51 as an example, two mutants (Hum51-M1 and Hum51-M2) were designed. In Hum51-M1, the sequence of the six nucleotides at the 3′-end was rearranged to make it lose the ninth GGG repeat. Thus, this mutant can fold into only a multimeric G-quadruplex structure with a linking loop of TTA. In Hum51-M2, the sequence between the 22nd and 30th nucleotide was rearranged. The loss of the fifth GGG repeat means this mutant can form only a multimeric G-quadruplex with a 9 nt linking loop. These mutants also have strand orientation identical with the wild-type under both dilute and molecular crowding conditions (Supplementary Figures S40 and S41), and they showed similar effects on the absorption and fluorescence spectra of *m*-TMPipEOPP and *p*-TMPipEOPP to the wild-type (Supplementary Figures S9 and S10). A *p*-TMPipEOPP/G-quadruplex binding stoichiometry of 1:1.6 was observed for the multimeric G-quadruplexes with the smallest pocket (Hum51-M1, Hum57-M1 and Hum63-M1, Table 2). This stoichiometry is identical with that obtained by Hum45, which might also fold into a multimeric G-quadruplex with a 3 nt linking loop. With the increase of the linking loop length to 9 nt (Hum51-M2, Hum57-M2 and Hum63-M2), the binding stoichiometry changed to 1:1.2, suggesting 20% *p*-TMPipEOPP adopted a sandwich-like end-stacking mode and the other 80% followed an intercalative mode. When the length of the linking loop between the two G-quadruplex units increased to 15 nt (the multimeric G-quadruplexes formed by Hum57-M3 and Hum63-M3), the binding stoichiometry increased to 1:1, indicating only the intercalative binding mode remained. These results suggest a big pocket is a benefit for the intercalative binding mode, and the pocket formed by a 15 nt linking loop is big enough for the intercalation of *p*-TMPipEOPP.

Hum69, which has 12 G-tracts, can fold into a trimeric G-quadruplex. It might exist also as a mixture of multimeric G-quadruplexes containing two G-quadruplex units linked by loops of different lengths. This G-rich sequence bound to *p*-TMPipEOPP with a stoichiometry of 1:1.

The binding stoichiometries of *m*-TMPipEOPP to monomeric and multimeric G-quadruplexes were determined under the same conditions. The absorption signals at wavelengths 452 and 666 nm were used to construct Job plots. As expected, no obvious inflection point was observed in the Job plots of Hum21, suggesting no obvious binding interaction occurred between *m*-TMPipEOPP and this monomeric G-quadruplex. This observation is consistent with the results of absorption and fluorescence spectroscopy mentioned above. By contrast, nearly identical stoichiometry (1:2) was obtained for Hum45, Hum51, Hum57 and Hum63, suggesting the pocket size had very little or nearly no effect on the binding interaction between *m*-TMPipEOPP and multimeric G-quadruplexes. One *m*-TMPipEOPP molecule might bind with two multimeric G-quadruplexes formed by these G-rich sequences. Scatchard analysis suggested that each multimeric G-quadruplex had 0.5 *m*-TMPipEOPP-binding site (Supplementary Figure S42, Supplementary Table S1), thus confirming the binding stoichiometry of 1:2 between *m*-TMPipEOPP and individual multimeric G-quadruplexes. The binding constant *K*_a_ was in the range of (1.05–2.53) × 10^6^ M^−1^, and there was no great difference between different multimeric G-quadruplexes.

According to the results of Job plot analysis, two binding modes could be supposed (Supplementary Scheme S1). One is the intercalative mode, in which one part of an *m*-TMPipEOPP molecule intercalates into the pocket of the first multimeric G-quadruplex, whereas the other part of the *m*-TMPipEOPP molecule intercalates into the pocket of the second multimeric G-quadruplex. Because neither of the two parts intercalates into the pockets deeply, a large pocket is not required; i.e. pocket size has little effect on intercalative binding. The other mode is side binding. In this mode, a sandwich-like G-quadruplex/*m*-TMPipEOPP/G-quadruplex complex is formed (Scheme 2), but the binding sites of *m*-TMPipEOPP are the side faces of G-quadruplexes rather than the ends of G-quadruplex units. It is quite likely *m*-TMPipEOPP is placed partly into the grooves of the G-quadruplexes (53–55). Such a binding mode has been reported for TMPyP4 and other pophyrin derivatives (35,56,57). Because the pocket is not the binding site of *m*-TMPipEOPP, its size is not relevant to the binding interaction. Both modes need four G-quadruplex units, which might increase the difficulty for interaction between *m*-TMPipEOPP and monomeric G-quadruplexes, because it requires the simultaneous proximity of four monomeric G-quadruplexes for one *m*-TMPipEOPP molecule. As a result, nearly no interaction was observed between *m*-TMPipEOPP and monomeric G-quadruplexes. By contrast, only two monomeric G-quadruplexes are required for the interaction between *p*-TMPipEOPP and monomeric G-quadruplexes. This is relatively easy and marked interaction between *p*-TMPipEOPP and monomeric G-quadruplexes was observed.

Hum69 might fold into a multimeric G-quadruplex containing three G-quadruplex units. Job plot analysis showed *m*-TMPipEOPP bound to Hum69 with a stoichiometry of 1:2. If *m*-TMPipEOPP binds to Hum69 by the intercalative mode, the proposed stoichiometry should be about 1:1, which is inconsistent with Job plot analysis. In addition, the different effects of multimeric G-quadruplexes on the fluorescence spectra of *m*-TMPipEOPP and *p*-TMPipEOPP suggested there should be different environments provided by multimeric G-quadruplexes to these two porphyrin isomers (Figures 3 and Supplementary Figure S10). Therefore, side binding might be the preferential binding mode between multimeric G-quadruplexes and *m*-TMPipEOPP. When the concentration of *m*-TMPipEOPP is much higher than that of multimeric G-quadruplex, a less obvious inflexion was observed in the Job plots constructed by *m*-TMPipEOPP and some multimeric G-quadruplexes, indicating that another binding mode might be occurred between them. This binding mode does not show identical binding stoichiometries for different multimeric G-quadruplexes, and it has little effect on the absorption signals of *m*-TMPipEOPP. Such a binding interaction might be attributed to the electrostatic attraction between cationic *m*-TMPipEOPP and anionic DNA molecule (58).

### G-quadruplex-stabilizing ability of m-TMPipEOPP

It was demonstrated *p*-TMPipEOPP could increase the stability of monomeric and multimeric G-quadruplexes in our earlier work (47). In this study, we found multimeric G-quadruplexes had weaker effects on the absorption and fluorescence spectra of *m*-TMPipEOPP compared to *p*-TMPipEOPP, implying the binding interaction between *m*-TMPipEOPP and multimeric G-quadruplexes might be weaker compared to *p*-TMPipEOPP and multimeric G-quadruplexes. To investigate whether *m*-TMPipEOPP could increase the stability of multimeric G-quadruplexes, the melting temperatures (*T*_1/2_) of G-quadruplexes in the absence or presence of *m*-TMPipEOPP were determined under both dilute and molecular crowding conditions. Corresponding experiments were done by recording the temperature-dependent absorbance decrease at wavelength 295 nm, which is caused by destruction of the G-quadruplex structure at elevated temperatures (59). The temperature at which the absorption signal is midway between the minimal and maximal levels is *T*_1/2_ (Supplementary Figures S43 and S44) (21). As shown in Table 3, the presence of *m*-TMPipEOPP had almost no effect on *T*_1/2_ of the monomeric G-quadruplex Hum21, confirming the above observation that no interaction occurs between *m*-TMPipEOPP and monomeric G-quadruplexes. By contrast, a marked stabilizing ability of *m*-TMPipEOPP to multimeric G-quadruplexes could be observed under both dilute and molecular crowding conditions, which was reflected by the increased *T*_1/2_ of multimeric G-quadruplexes in the presence of *m*-TMPipEOPP. The effect of *m*-TMPipEOPP on *T*_1/2_ values of multimeric G-quadruplexes under molecular crowding conditions was not so great as that seen under dilute conditions, which is consistent with the previous report on monomeric G-quadruplex ligands (46). One reason might be the increased stability of multimeric G-quadruplexes themselves under molecular crowding conditions. The effect of *p*-TMPipEOPP on multimeric G-quadruplex stability was studied in our earlier work (47). We found the change of *T*_1/2_ caused by *m*-TMPipEOPP was a little less compared to *p*-TMPipEOPP, indicating the multimeric G-quadruplex-stabilizing ability of *m*-TMPipEOPP is weaker compared to *p*-TMPipEOPP, but *m*-TMPipEOPP has better multimeric G-quadruplex recognition specificity compared to *p*-TMPipEOPP.

**Table 3.** G-quadruplex-stabilizing ability of *m*-TMPipEOPP

	*T*_1/2_ under dilute conditions (°C)		*T*_1/2_ under molecular crowding conditions (°C)	
G-quadruplexes	[*m*-TMPipEOPP] (μM)		[*m*-TMPipEOPP] (μM)	
	0	5	0	5
Hum21	60.4	60.8	70.6	69.6
Hum45	45.8	54.9	51.5	56.6
Hum51	47.6	55.6	53.1	57.9
Hum57	49.8	59.0	56.0	62.4
Hum63	51.4	60.3	57.4	63.4
Hum69	48.6	55.2	52.2	58.1

### Effects of pocket size on the stability of multimeric G-quadruplex

Another interesting phenomenon is that pocket size can affect the stability of multimeric G-quadruplex (Supplementary Figures S45 and S46). Under both dilute and molecular crowding conditions, monomeric Hum21 showed higher *T*_1/2_ values compared to multimeric G-quadruplexes (Table 4), suggesting monomeric are more stable compared to multimeric G-quadruplexes formed by similar G-rich sequences. DNA sequence length-dependent *T*_1/2_ increase was observed for the multimeric G-quadruplexes formed by Hum45, Hum 51, Hum57 and Hum63, suggesting multimeric G-quadruplex with a large pocket should have a high level of stability. Hum69 possibly has the ability to fold into a multimeric G-quadruplex containing three G-quadruplex units and two TTA linking loops; such a multimeric G-quadruplex with small pockets should have a relatively low level of stability. The experimental results support such a supposition. Hum69 gave lower *T*_1/2_ values compared to Hum57 and Hum63 under both dilute and molecular crowding conditions.

**Table 4.** Stabilities of multimeric G-quadruplexes with different pocket sizes

	*T*_1/2_ (°C)			*T*_1/2_ (°C)	
G-quadruplex	Dilute conditions	Molecular crowding conditions	G-quadruplex	Dilute conditions	Molecular crowding conditions
Hum21	60.4	70.6	Hum57-M1	45.6	50.7
Hum45	45.8	51.5	Hum63-M1	45.8	51.5
Hum51	47.6	53.1	Hum51-M2	51.3	55.2
Hum57	49.8	56.0	Hum57-M2	51.4	55.0
Hum63	51.4	57.4	Hum63-M2	51.4	54.8
Hum69	48.6	52.2	Hum57-M3	52.6	57.5
Hum51-M1	45.8	51.4	Hum63-M3	52.6	57.6

In the experiments described above, we designed several mutants of Hum51, Hum57 and Hum63 to form multimeric G-quadruplexes with definite sizes of pocket. To further demonstrate the effect of pocket size on G-quadruplex stability, *T*_1/2_ values of the multimeric G-quadruplexes formed by these mutants were compared. Under both dilute and molecular crowding conditions, a pocket size-dependent *T*_1/2_ increase was observed for the multimeric G-quadruplexes formed by these mutants. Take Hum51, Hum51-M1 and Hum51-M2 as example; Hum52-M1 gave the minimum and Hum52-M2 gave the maximum *T*_1/2_. Hum51 gave a *T*_1/2_ value between those of Hum51-M1 and Hum51-M2, suggesting Hum51 might form a mixture of two multimeric G-quadruplexes with pockets of different sizes. Interestingly, similar *T*_1/2_ values were obtained when multimeric G-quadruplexes with the same size of pocket were formed (e.g. the multimeric G-quadruplexes formed by Hum51-M1, Hum57-M1 and Hum63-M1). As we expected, the size of the pocket is an important factor in determining the stability of multimeric G-quadruplex. Such a pocket size-dependent increase of multimeric G-quadruplex stability gives support for the proposed binding mechanism between *p*-TMPipEOPP and multimeric G-quadruplexes.

## CONCLUSION

In summary, a new cationic porphyrin derivative *m*-TMPipEOPP, with positively charged side arm substituents at the meta-position of benzene, was synthesized, characterized and its multimeric G-quadruplex recognition specificity under molecular crowding conditions was compared with its isomer *p*-TMPipEOPP, with positively charged side arm substituents at the para-position of benzene. The slight structural difference accounts for the markedly different multimeric G-quadruplex recognition specificities of the two porphyrin derivatives. *p*-TMPipEOPP has nearly no ability to discriminate between multimeric and monomeric G-quadruplexes. The stoichiometry of 1:2 suggests *p*-TMPipEOPP might bind to monomeric G-quadruplexes by a sandwich-like end-stacking mode. The binding stoichiometry between *p*-TMPipEOPP and multimeric G-quadruplex is affected greatly by the size of pocket between two adjacent G-quadruplex units. Their binding interactions might follow two modes: sandwich-like end-stacking mode and the intercalation of *p*-TMPipEOPP into the pocket between two G-quadruplex units. The increase of pocket size is a benefit for the intercalative mode. On the contrary, *m*-TMPipEOPP could not interact with monomeric G-quadruplexes at all, and a relatively constant stoichiometry, which is nearly unaffected by the pocket size, was observed for the interaction between *m*-TMPipEOPP and multimeric G-quadruplexes. The stoichiometry of two multimeric G-quadruplexes per *m*-TMPipEOPP molecule suggests *m*-TMPipEOPP might bind to multimeric G-quadruplexes by a side binding mode. Such a binding mode confers *m*-TMPipEOPP with higher multimeric G-quadruplex recognition specificity compared to *p*-TMPipEOPP. To our knowledge, this is the first example of a ligand that can specifically distinguish multimeric G-quadruplexes from monomeric ones under molecular crowding conditions. These results might provide important information for the design of highly specific multimeric G-quadruplex ligands. Under both dilute and molecular crowding conditions, the presence of *m*-TMPipEOPP could increase the stability of a multimeric G-quadruplex, although its multimeric G-quadruplex-stabilizing ability was a little lower compared to its isomer *p*-TMPipEOPP. Another interesting finding in this work is that pocket size is an important factor in determining the stability of multimeric G-quadruplexes. Our next work is to design highly specific multimeric G-quadruplex ligands with enhanced G-quadruplex-stabilizing ability on the basis of the observations reported here.

## SUPPLEMENTARY DATA

Supplementary Data are available at NAR Online.

SUPPLEMENTARY DATA
