# Supplementary material for: Two cationic porphyrin isomers showing different multimeric G-quadruplex recognition specificity against monomeric G-quadruplexes
Source: Nucleic Acids Res. 2014 Jun 17;42(13):8719–31. doi: 10.1093/nar/gku526 (PMC4117758; doi:10.1093/nar/gku526)
Supplement: SUPPLEMENTARY DATA [file supp_42_13_8719__index.html]

Two cationic porphyrin isomers showing different multimeric G-quadruplex recognition specificity against monomeric G-quadruplexes — SUPPLEMENTARY DATA 

# Two cationic porphyrin isomers showing different multimeric G-quadruplex recognition specificity against monomeric G-quadruplexes

## SUPPLEMENTARY DATA

**Files in this Data Supplement:**

- SUPPLEMENTARY DATA
