# Supplementary material for: Two cationic porphyrin isomers showing different multimeric G-quadruplex recognition specificity against monomeric G-quadruplexes
Source: Nucleic Acids Res. 2014 Jun 17;42(13):8719–31. doi: 10.1093/nar/gku526 (PMC4117758; doi:10.1093/nar/gku526)
Supplement: SUPPLEMENTARY DATA [file supp_gku526_nar-00741-f-2014-File002.docx]

Supporting information for

Two cationic porphyrin isomers showing different multimeric G-quadruplex recognition specificity against monomeric G-quadruplexes

Xiao-Xi Huang^1,2^, Li-Na Zhu^2,3^, Bin Wu^3^, Yan-Fang Huo^3^, Na-Na Duan^1,2^ and De-Ming Kong^1,2,^*

^1^ State Key Laboratory of Medicinal Chemical Biology, Nankai University, Tianjin, 300071, P R China

^2^ Collaborative Innovation Center of Chemical Science and Engineering (Tianjin), Tianjin 300071, P R China

^3^ Department of Chemistry, Tianjin University, Tianjin, 300072, P R China

* Corresponding authors: De-Ming Kong. Tel: (+)86-22-23500938; Fax: (+)86-22-23502458; Email: kongdem@nankai.edu.cn

1. **Synthesis** **and Characterization of 5,10,15,20-tetra-{3-[2-(1-methyl-1- piperidinyl) ethoxy] phenyl} porphyrin tetraiodide (*m*-TMPipEOPP**)

**1.1 Synthesis and characterization of 5,10,15,20-tetrakis (3-hydroxyphenyl) porphyrin (THPP).**

A suspension of 3-Hydroxybenzaldehyde (6.1 g, 0.05 mol) was dissolved in 100 mL propionic acid/DMSO (dimethyl sulfoxide) (v:v = 47:3) and the mixture was stirred at 128 ^o^C. Then, 3.5 mL (0.05 mol) of freshly prepared pyrrole was added. The mixture was heated to 141 ^o^C and refluxed for 2h, then, cooled to room temperature and filtrated under reduced pressure. The crude residue was redissolved in CH_2_Cl_2_ or CH_2_Cl_2_/CH_3_OH mixture, and purified using silica gel columns (100–200 mesh). CH_2_Cl_3_/MeOH mixture (v:v = 7:1) was used to elute the pure product. The purple solid of the product was obtained in 7.04% yield (0.5978g, 0.88 mmol). ^1^H NMR (300 MHz, [D_6_]DMSO, 25 ºC, TMS) (Figure S1): δ = 9.89 (s, 4H; phenolic hydroxyl H), 8.86 (s, 8H; β-pyrrole H), 7.53 (s, 12 H; Ph-H), 7.23 (s, 2H; Ph-H), 7.20 (s, 2H; Ph-H), -2.99 ppm (s, 2H; pyrrole H); ESI:m/z: calcd for C_44_H_30_N_4_O_4_[M+H^+^]: 679.23; found: 679.64[M+H^+^]. FT-MS: m/z: calcd for C_44_H_30_N_4_O_4_: 679.23398; found: 679.2338[M].


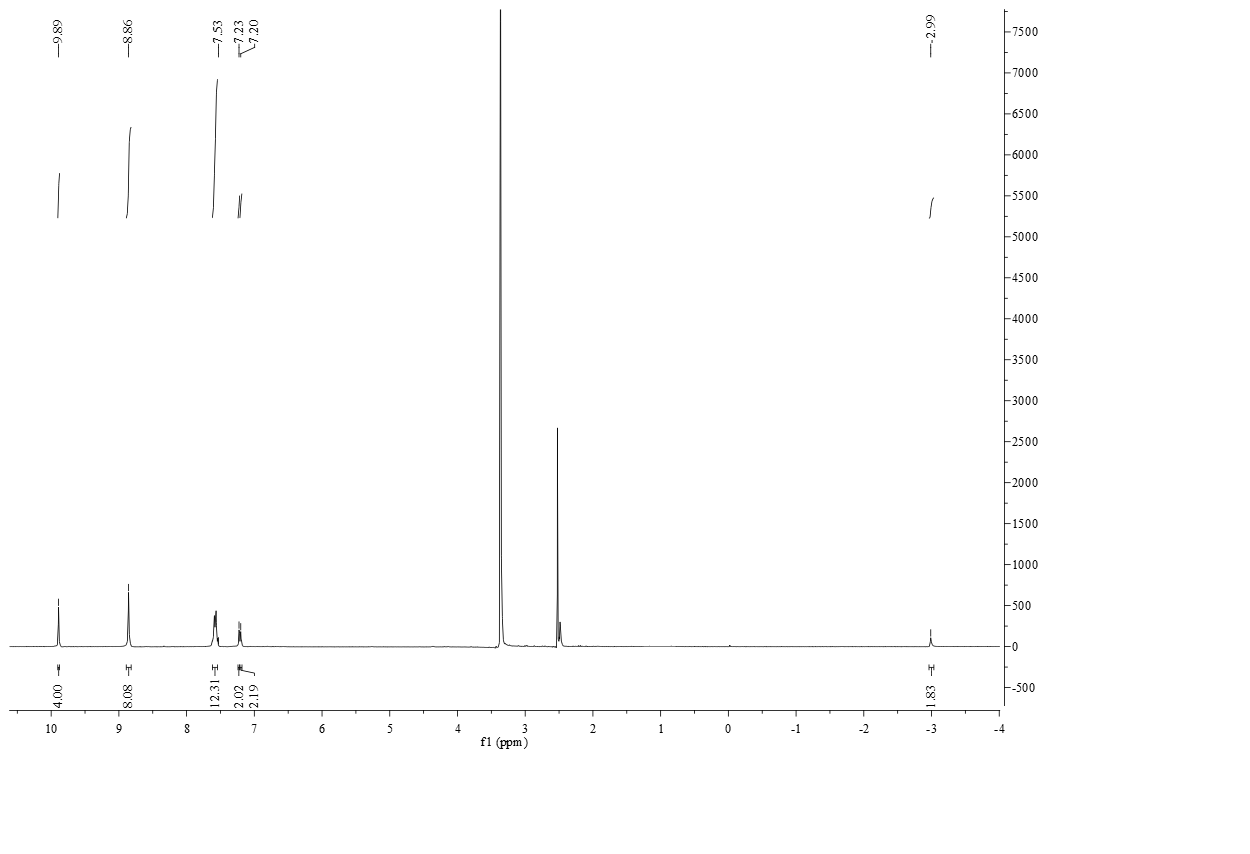


**Figure S1**. ^1^H-NMR of 5,10,15,20-tetrakis(3-hydroxyphenyl)porphyrin (THPP). The NMR spectra were recorded on Mercury Vx-300 spectrometer operating for ^1^H NMR. Chemical shifts in the ^1^H NMR spectra are reported in ppm relative to the residual hydrogen atoms in the deuterated solvents: d = 2.50 and 7.25 ppm for [D_6_]DMSO and CDCl_3_, respectively.

**1.2 Synthesis and characterization of 5,10,15,20-tetra-{3-[2-(1-piperidinyl) ethoxy]phenyl} porphyrin (TPipEOPP).**


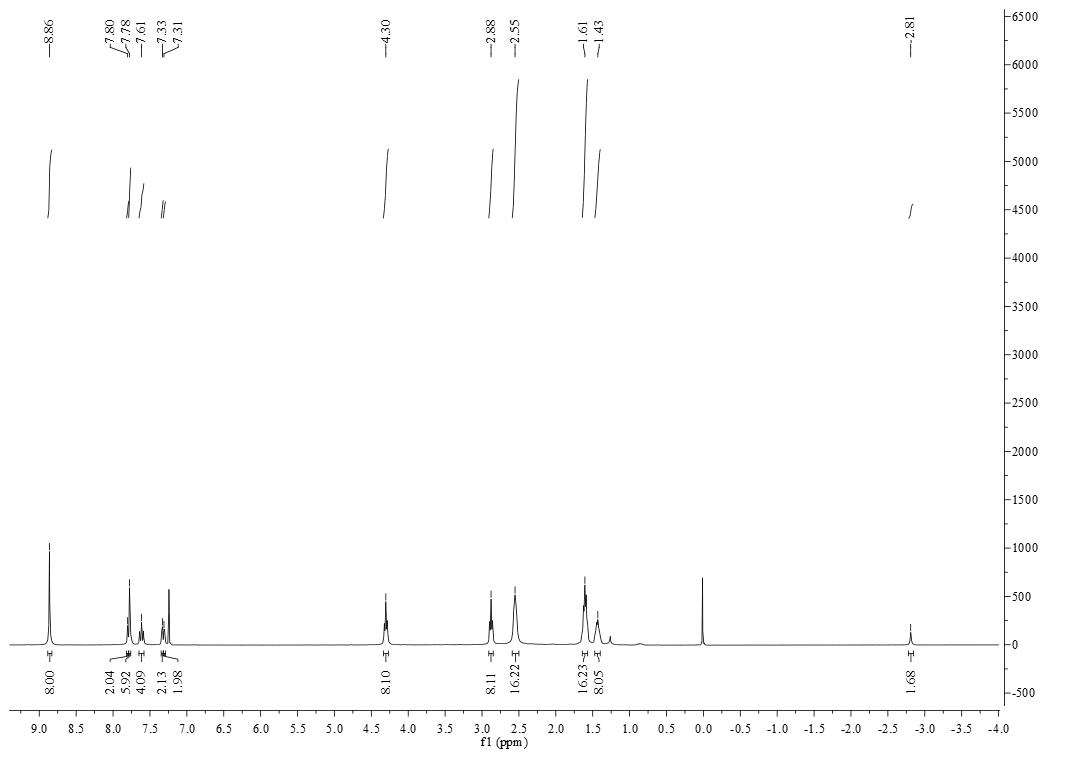
A suspension of 5,10,15,20-tetrakis (3-hydroxyphenyl) porphyrin (THPP) (0.2172g, 0.32 mmol), 1-(2-chloroethyl)-piperidine hydrochloride (0.4714 g, 2.56 mmol), and K_2_CO_3_ (0.6192g, 4.48 mmol) in dry DMF (50 mL) was stirred for 72 h at room temperature under N_2_. Then the mixture was filtered. The red-brown precipitate was obtained and washed with DMF (10 mL) and diethyl ether (5 mL). The residue was dissolved in dichloromethane (100 mL) and washed with water. The organic layer was evaporated under reduced pressure. The resulting solid was isolated by chromatography on alumina (100-200 mesh) with ethyl acetate /methanol (v:v = 50:1). The first fraction was collected and the solvent was evaporated. Further purification was carried out by recrystallization from CH_3_OH/CH_2_Cl_2_ (v:v = 20:1). The purple-brown crystals of the product TPipEOPP were obtained in 56.9% yield (0.124 g, 0.11 mmol). ^1^H NMR (300 MHz, CDCl_3_, 25 ^o^C, TMS) (Figure S2): δ = 8.86 (s, 8H; β-pyrrole H), 7.80 (s, 2H; Ph-H), 7.78 (s, 6H; Ph-H), 7.61 (s, 4H; Ph-H), 7.33 (s, 2H; Ph-H), 7.31 (s, 2H; Ph-H), 4.30 (s, 8H; OCH_2_), 2.88 (s, 8H; NCH_2_), 2.55 (s, 16H; piperidine H), 1.61 (s, 16H; piperidine H), 1.43 (s, 8H; piperidineH), -2.81 ppm (s, 2H; pyrrole H); ESI:m/z: calcd for C_72_H_81_N_8_O_4_[M+H^+^]: 1123.47; found:1124.06[M+H^+^]. FT-MS: m/z: calcd for C_72_H_81_N_8_O_4_: 1123.65318; found: 1123.6530[M].

**Figure S2**. ^1^H-NMR of 5,10,15,20-tetra- {3-[2-(1-piperidinyl)ethoxy]phenyl} porphyrin (TPipEOPP). The NMR spectra were recorded on Mercury Vx-300 spectrometer operating for ^1^H NMR. Chemical shifts in the ^1^H NMR spectra are reported in ppm relative to the residual hydrogen atoms in the deuterated solvents: d = 2.50 and 7.25 ppm for [D_6_]DMSO and CDCl_3_, respectively.

**1.3 Synthesis and characterization of 5,10,15,20-tetra-{3-[2-(1-methyl-1- piperidinyl) ethoxy] phenyl} porphyrin tetraiodide (*m*-TMPipEOPP-4I**).

To a suspension of TPipEOPP (0.0584g, 0.052 mmol) in dry CH_2_Cl_2_ (35 mL) was added CH_3_I (15 mL, 0.24 mmol). The mixture was stirred under N_2_ and heated by using an oil bath at 40 ^o^C for 24 h. The solvent was evaporated and the resulting solid was washed with CH_2_Cl_2_ and diethyl ether in turn. *m*-TMPipEOPP-4I was obtained as a red-purple solid in 60.7% yield (0.0355 g, 0.030 mmol). ^1^H NMR (300 MHz, [D_6_]DMSO, 25 ºC, TMS) (Figure S1): δ = 8.90 (s, 8H; β-pyrrole H), 7.84 (s, 8H; Ph-H), 7.78 (s, 4H; Ph-H), 7.52 (s, 4H; Ph-H), 4.69 (s, 8H; OCH_2_), 3.92 (s, 8H; NCH_2_), 3.41–3.33 (m, 16H; piperidine H), 3.19 (s, 12H; NCH_3_), 2.49 (s, 8H; piperidine H), 1.84 (s, 16H; piperidine H), -2.95 ppm (s, 2H; pyrrole H); ESI:m/z: calcd for [C_76_H_93_N_8_O_4_-4I]/4: 295.7; found: 296.2 [M^+^-4I]/4. FT-MS: m/z: calcd for [C_76_H _93_N_8_ O_4_-4I]/4: 295.68440; found: 295.6847[M].


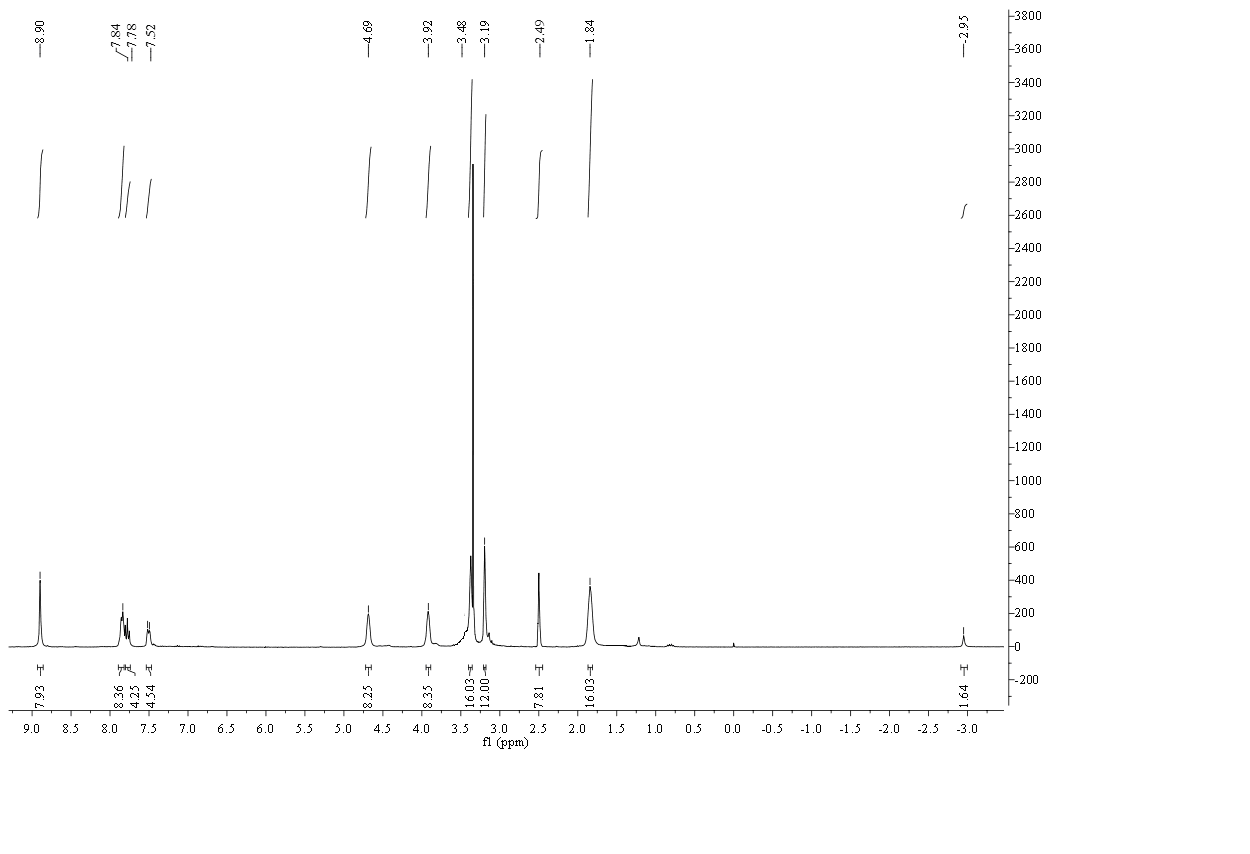


**Figure S3**. ^1^H-NMR of 5,10,15,20-tetra- {3-[2-(1-methyl-1-piperidinyl)ethoxy]phenyl} porphyrin (*m*-TMPipEOPP). The NMR spectra were recorded on Mercury Vx-300 spectrometer operating for ^1^H NMR. Chemical shifts in the ^1^H NMR spectra are reported in ppm relative to the residual hydrogen atoms in the deuterated solvents: d = 2.50 and 7.25 ppm for [D_6_]DMSO and CDCl_3_, respectively.

1. **Effects of different DNAs on the UV-vis absorption spectrum of** *m*-TMPipEOPP

Figure S4. UV-vis absorption spectra of *m*-TMPipEOPP in absence or presence of different DNAs. Free porphyrin (black line); G-quadruplex (red line); duplex DNA (blue line); single-stranded DNA (green line). [porphyrin] = 2.5 μM; [multimeric quadruplex] = 10 μM; [duplex DNA] = [single-stranded DNA] = 20 μM.

1. **DNA concentration-dependent changes in the absorption spectrum of** *m*-TMPipEOPP
   1. **Duplex DNAs**

Figure S5. DNA concentration-dependent absorption spectrum changes of *m*-TMPipEOPP in the presence of individual duplex DNAs. The concentrations of the DNAs are labelled in the figures.

**3.2 Single-stranded DNA**

Figure S6. DNA concentration-dependent absorption spectrum changes of *m*-TMPipEOPP in the presence of single-stranded ssDNA2. The concentrations of ssDNA2 are labelled in the figure.

**3.3 Multimeric G-quadruplexes**

Figure S7. DNA concentration-dependent absorption spectrum changes of *m*-TMPipEOPP in the presence of individual multimeric G-quadurplexes. The concentrations of the G-quadruplexes are (arrow direction): 0, 2.5, 5, 10, 15 and 20 μM.

**3.4 Monomeric G-quadruplexes**

Figure S8. DNA concentration-dependent absorption spectrum changes of *m*-TMPipEOPP in the presence of individual monomeric G-quadurplexes (M3Q, Oxy28, C-MYC and KRAS). The concentrations of the G-quadruplexes are labelled in the figures.

**3.5 Mutants of multimeirc G-quadruplexes**

Figure S9. DNA concentration-dependent absorption spectrum changes of *m*-TMPipEOPP in the presence of the mutants of multimeric G-quadurplexes. The concentrations of the G-quadruplexes are (arrow direction): 0, 2.5, 5, 10, 15 and 20 μM.

1. **DNA concentration-dependent fluorescence spectral changes of the two porphyrin isomers in the presence of multimeric G-quadruplexes or their mutants**

Figure S10. DNA concentration-dependent fluorescence spectrum changes of *m*-TMPipEOPP (Left) and *p*-TMPipEOPP (Right) in the presence of multimeric G-quadurplexes and their mutants. When *m*-TMPipEOPP was used, the concentrations of the G-quadruplexes are (arrow direction): 0, 1, 2, 3, 4, 5, 6, 7, 8, 9, 10, 15, 20 and 25 μM. When *p*-TMPipEOPP was used, the concentrations of the G-quadruplexes are (arrow direction): 0, 0.1, 0.2, 0.3, 0.4, 0.5, 0.6, 0.7, 0.8, 0.9, 1, 1.5, 2, 2.5, 3, 3.5, 4, 5, 6, 7, 8, 9, 10, 15, 20 and 25 μM.

Figure S11. Comparison of fluorescence signal changes (F_674nm_-F_709nm_) in the presence of multimeric G-quadruplex Hum45 and monomeric G-quadruplex Hum21.

1. Job Plot analysis for the interactions between *p*-TMPipEOPP and G-quadruplexes
   1. Monomeric G-quadruplex Hum21

Figure S12. Job plot analysis of the interaction between *p*-TMPipEOPP and Hum21 utilizing the absorption signals at (a) 421 nm, (b) 454 nm, (c) 695 nm and (d) the fluorescence signals at 719 nm (λ_ex_ = 700 nm), respectively. (a) [*p*-TMPipEOPP] + [Hum21] = 2 μM, (b) and (d) [*p*-TMPipEOPP] + [Hum21] = 5 μM, (c) [*p*-TMPipEOPP] + [Hum21] = 30 μM.

- 1. Multimeric G-quadruplex Hum45

Figure S13. Job plot analysis of the interaction between *p*-TMPipEOPP and Hum45 utilizing the absorption signals at (a) 421 nm, (b) 454 nm, (c) 695 nm and (d) the fluorescence signals at 719 nm (λ_ex_ = 700 nm), respectively. (a) [*p*-TMPipEOPP] + [Hum45] = 2 μM, (b) and (d) [*p*-TMPipEOPP] + [Hum45] = 5 μM, (c) [*p*-TMPipEOPP] + [Hum45] = 30 μM.

- 1. Multimeric G-quadruplex Hum51

Figure S14. Job plot analysis of the interaction between *p*-TMPipEOPP and Hum51 utilizing the absorption signals at (a) 421 nm, (b) 454 nm, (c) 695 nm and (d) the fluorescence signals at 719 nm (λ_ex_ = 700 nm), respectively. (a) [*p*-TMPipEOPP] + [Hum51] = 2 μM, (b) and (d) [*p*-TMPipEOPP] + [Hum51] = 5 μM, (c) [*p*-TMPipEOPP] + [Hum51] = 30 μM.

- 1. Multimeric G-quadruplex Hum57

Figure S15. Job plot analysis of the interaction between *p*-TMPipEOPP and Hum57 utilizing the absorption signals at (a) 421 nm, (b) 454 nm, (c) 695 nm and (d) the fluorescence signals at 719 nm (λ_ex_ = 700 nm), respectively. (a) [*p*-TMPipEOPP] + [Hum57] = 2 μM, (b) and (d) [*p*-TMPipEOPP] + [Hum57] = 5 μM, (c) [*p*-TMPipEOPP] + [Hum57] = 30 μM.

- 1. Multimeric G-quadruplex Hum63

Figure S16. Job plot analysis of the interaction between *p*-TMPipEOPP and Hum63 utilizing the absorption signals at (a) 421 nm, (b) 454 nm, (c) 695 nm and (d) the fluorescence signals at 719 nm (λ_ex_ = 700 nm), respectively. (a) [*p*-TMPipEOPP] + [Hum63] = 2 μM, (b) and (d) [*p*-TMPipEOPP] + [Hum63] = 5 μM, (c) [*p*-TMPipEOPP] + [Hum63] = 30 μM.

- 1. Multimeric G-quadruplex Hum69

Figure S17. Job plot analysis of the interaction between *p*-TMPipEOPP and Hum69 utilizing the absorption signals at (a) 421 nm, (b) 454 nm, (c) 695 nm and (d) the fluorescence signals at 719 nm (λ_ex_ = 700 nm), respectively. (a) [*p*-TMPipEOPP] + [Hum69] = 2 μM, (b) and (d) [*p*-TMPipEOPP] + [Hum69] = 5 μM, (c) [*p*-TMPipEOPP] + [Hum69] = 30 μM.

- 1. Multimeric G-quadruplex Hum51-M1

Figure S18. Job plot analysis of the interaction between *p*-TMPipEOPP and Hum51-M1 utilizing the absorption signals at (a) 421 nm, (b) 454 nm, (c) 695 nm and (d) the fluorescence signals at 719 nm (λ_ex_ = 700 nm), respectively. (a) [*p*-TMPipEOPP] + [Hum51-M1] = 2 μM, (b) and (d) [*p*-TMPipEOPP] + [Hum51-M1] = 5 μM, (c) [*p*-TMPipEOPP] + [Hum51-M1] = 30 μM.

- 1. Multimeric G-quadruplex Hum57-M1

Figure S19. Job plot analysis of the interaction between *p*-TMPipEOPP and Hum57-M1 utilizing the absorption signals at (a) 421 nm, (b) 454 nm, (c) 695 nm and (d) the fluorescence signals at 719 nm (λ_ex_ = 700 nm), respectively. (a) [*p*-TMPipEOPP] + [Hum57-M1] = 2 μM, (b) and (d) [*p*-TMPipEOPP] + [Hum57-M1] = 5 μM, (c) [*p*-TMPipEOPP] + [Hum57-M1] = 30 μM.

- 1. Multimeric G-quadruplex Hum63-M1

Figure S20. Job plot analysis of the interaction between *p*-TMPipEOPP and Hum63-M1 utilizing the absorption signals at (a) 421 nm, (b) 454 nm, (c) 695 nm and (d) the fluorescence signals at 719 nm (λ_ex_ = 700 nm), respectively. (a) [*p*-TMPipEOPP] + [Hum63-M1] = 2 μM, (b) and (d) [*p*-TMPipEOPP] + [Hum63-M1] = 5 μM, (c) [*p*-TMPipEOPP] + [Hum63-M1] = 30 μM.

- 1. Multimeric G-quadruplex Hum51-M2

Figure S21. Job plot analysis of the interaction between *p*-TMPipEOPP and Hum51-M2 utilizing the absorption signals at (a) 421 nm, (b) 454 nm, (c) 695 nm and (d) the fluorescence signals at 719 nm (λ_ex_ = 700 nm), respectively. (a) [*p*-TMPipEOPP] + [Hum51-M2] = 2 μM, (b) and (d) [*p*-TMPipEOPP] + [Hum51-M2] = 5 μM, (c) [*p*-TMPipEOPP] + [Hum51-M2] = 30 μM.

- 1. Multimeric G-quadruplex Hum57-M2

Figure S22. Job plot analysis of the interaction between *p*-TMPipEOPP and Hum57-M2 utilizing the absorption signals at (a) 421 nm, (b) 454 nm, (c) 695 nm and (d) the fluorescence signals at 719 nm (λ_ex_ = 700 nm), respectively. (a) [*p*-TMPipEOPP] + [Hum57-M2] = 2 μM, (b) and (d) [*p*-TMPipEOPP] + [Hum57-M2] = 5 μM, (c) [*p*-TMPipEOPP] + [Hum57-M2] = 30 μM.

- 1. Multimeric G-quadruplex Hum63-M2

Figure S23. Job plot analysis of the interaction between *p*-TMPipEOPP and Hum63-M2 utilizing the absorption signals at (a) 421 nm, (b) 454 nm, (c) 695 nm and (d) the fluorescence signals at 719 nm (λ_ex_ = 700 nm), respectively. (a) [*p*-TMPipEOPP] + [Hum63-M2] = 2 μM, (b) and (d) [*p*-TMPipEOPP] + [Hum63-M2] = 5 μM, (c) [*p*-TMPipEOPP] + [Hum63-M2] = 30 μM.

- 1. Multimeric G-quadruplex Hum57-M3

Figure S24. Job plot analysis of the interaction between *p*-TMPipEOPP and Hum57-M3 utilizing the absorption signals at (a) 421 nm, (b) 454 nm, (c) 695 nm and (d) the fluorescence signals at 719 nm (λ_ex_ = 700 nm), respectively. (a) [*p*-TMPipEOPP] + [Hum57-M3] = 2 μM, (b) and (d) [*p*-TMPipEOPP] + [Hum57-M3] = 5 μM, (c) [*p*-TMPipEOPP] + [Hum57-M3] = 30 μM.

- 1. Multimeric G-quadruplex Hum63-M3

Figure S25. Job plot analysis of the interaction between *p*-TMPipEOPP and Hum63-M3 utilizing the absorption signals at (a) 421 nm, (b) 454 nm, (c) 695 nm and (d) the fluorescence signals at 719 nm (λ_ex_ = 700 nm), respectively. (a) [*p*-TMPipEOPP] + [Hum63-M3] = 2 μM, (b) and (d) [*p*-TMPipEOPP] + [Hum63-M3] = 5 μM, (c) [*p*-TMPipEOPP] + [Hum63-M3] = 30 μM.

1. Job Plot analysis for the interactions between *m*-TMPipEOPP and G-quadruplexes

6.1 Monomeric G-quadruplex Hum21

Figure S26. Job plot analysis of the interaction between *m*-TMPipEOPP and Hum21 utilizing the absorption signals at 452 nm and 666 nm, respectively. [*m*-TMPipEOPP] + [Hum21] = 10 μM.

6.2 Multimeric G-quadruplex Hum45

Figure S27. Job plot analysis of the interaction between *m*-TMPipEOPP and Hum45 utilizing the absorption signals at 452 nm and 666 nm, respectively. [*m*-TMPipEOPP] + [Hum45] = 10 μM.

6.3 Multimeric G-quadruplex Hum51

Figure S28. Job plot analysis of the interaction between *m*-TMPipEOPP and Hum51 utilizing the absorption signals at 452 nm and 666 nm, respectively. [*m*-TMPipEOPP] + [Hum51] = 10 μM.

6.4 Multimeric G-quadruplex Hum57

Figure S29. Job plot analysis of the interaction between *m*-TMPipEOPP and Hum57 utilizing the absorption signals at 452 nm and 666 nm, respectively. [*m*-TMPipEOPP] + [Hum57] = 10 μM.

6.5 Multimeric G-quadruplex Hum63

Figure S30. Job plot analysis of the interaction between *m*-TMPipEOPP and Hum63 utilizing the absorption signals at 452 nm and 666 nm, respectively. [*m*-TMPipEOPP] + [Hum63] = 10 μM.

6.6 Multimeric G-quadruplex Hum69

Figure S31. Job plot analysis of the interaction between *m*-TMPipEOPP and Hum69 utilizing the absorption signals at 452 nm and 666 nm, respectively. [*m*-TMPipEOPP] + [Hum69] = 10 μM.

6.7 Multimeric G-quadruplex Hum51-M1

Figure S32. Job plot analysis of the interaction between *m*-TMPipEOPP and Hum51-M1 utilizing the absorption signals at 452 nm and 666 nm, respectively. [*m*-TMPipEOPP] + [Hum51-M1] = 10 μM.

6.8 Multimeric G-quadruplex Hum57-M1

Figure S33. Job plot analysis of the interaction between *m*-TMPipEOPP and Hum57-M1 utilizing the absorption signals at 452 nm and 666 nm, respectively. [*m*-TMPipEOPP] + [Hum57-M1] = 10 μM.

6.9 Multimeric G-quadruplex Hum63-M1

Figure S34. Job plot analysis of the interaction between *m*-TMPipEOPP and Hum63-M1 utilizing the absorption signals at 452 nm and 666 nm, respectively. [*m*-TMPipEOPP] + [Hum63-M1] = 10 μM.

6.10 Multimeric G-quadruplex Hum51-M2

Figure S35. Job plot analysis of the interaction between *m*-TMPipEOPP and Hum51-M2 utilizing the absorption signals at 452 nm and 666 nm, respectively. [*m*-TMPipEOPP] + [Hum51-M2] = 10 μM.

6.11 Multimeric G-quadruplex Hum57-M2

Figure S36. Job plot analysis of the interaction between *m*-TMPipEOPP and Hum57-M2 utilizing the absorption signals at 452 nm and 666 nm, respectively. [*m*-TMPipEOPP] + [Hum57-M2] = 10 μM.

6.12 Multimeric G-quadruplex Hum63-M2

Figure S37. Job plot analysis of the interaction between *m*-TMPipEOPP and Hum63-M2 utilizing the absorption signals at 452 nm and 666 nm, respectively. [*m*-TMPipEOPP] + [Hum63-M2] = 10 μM.

6.13 Multimeric G-quadruplex Hum57-M3

Figure S38. Job plot analysis of the interaction between *m*-TMPipEOPP and Hum57-M3 utilizing the absorption signals at 452 nm and 666 nm, respectively. [*m*-TMPipEOPP] + [Hum57-M3] = 10 μM.

6.14 Multimeric G-quadruplex Hum63-M3

Figure S39. Job plot analysis of the interaction between *m*-TMPipEOPP and Hum63-M3 utilizing the absorption signals at 452 nm and 666 nm, respectively. [*m*-TMPipEOPP] + [Hum63-M3] = 10 μM.

1. **Circular dichroism (CD) spectroscopy of monomeric and multimeric G-quadruplexes**
   1. Under dilute conditions

Figure S40. CD spectra of monomeric and multimeric G-quadruplexes under dilute conditions

7.2 Under molecular crowding conditions

Figure S41. CD spectra of monomeric and multimeric G-quadruplexes under molecular crowding conditions

1. Scatchard analysis of the interactions between ***m*-TMPipEOPP** and multimeric G-quadruplexes

**Figure S42**. Scatchard plots for *m*-TMPipEOPP with multimeric G-quadruplexes. The change in the absorbance difference between 452 and 418 nm as a function of G-quadruplex concentration was used to construct Scatchard plots

**Table S1**. Binding parameters for the interactions between *m*-TMPipEOPP and multimeric G-quadruplexes

| G-quadruplex | *n^a^* | *K*_a_  (×10^-6^ M^-1^)*^b^* | G-quadruplex | *n^a^* | *K*_a_  (×10^-6^ M^-1^)*^b^* |
| --- | --- | --- | --- | --- | --- |
| Hum 21 | undetected | undetected | Hum 51-M2 | 0.46 | 1.10 |
| Hum45 | 0.46 | 1.11 | Hum 57-M1 | 0.48 | 1.30 |
| Hum51 | 0.50 | 1.66 | Hum 57-M2 | 0.47 | 1.38 |
| Hum57 | 0.48 | 1.05 | Hum 57-M3 | 0.50 | 1.08 |
| Hum63 | 0.52 | 2.53 | Hum 63-M1 | 0.46 | 2.08 |
| Hum69 | 0.52 | 2.05 | Hum 63-M2 | 0.50 | 1.10 |
| Hum 51-M1 | 0.45 | 1.09 | Hum 63-M3 | 0.50 | 1.08 |

*^a^n* is the number of *m*-TMPipEOPP-binding sites on the G-quadruplex

*^b^K*_a_ is the binding constant for the interaction between *m*-TMPipEOPP and multimeric G-quadurplexes

1. Two possible binding modes between ***m*-TMPipEOPP** and multimeric G-quadruplexes


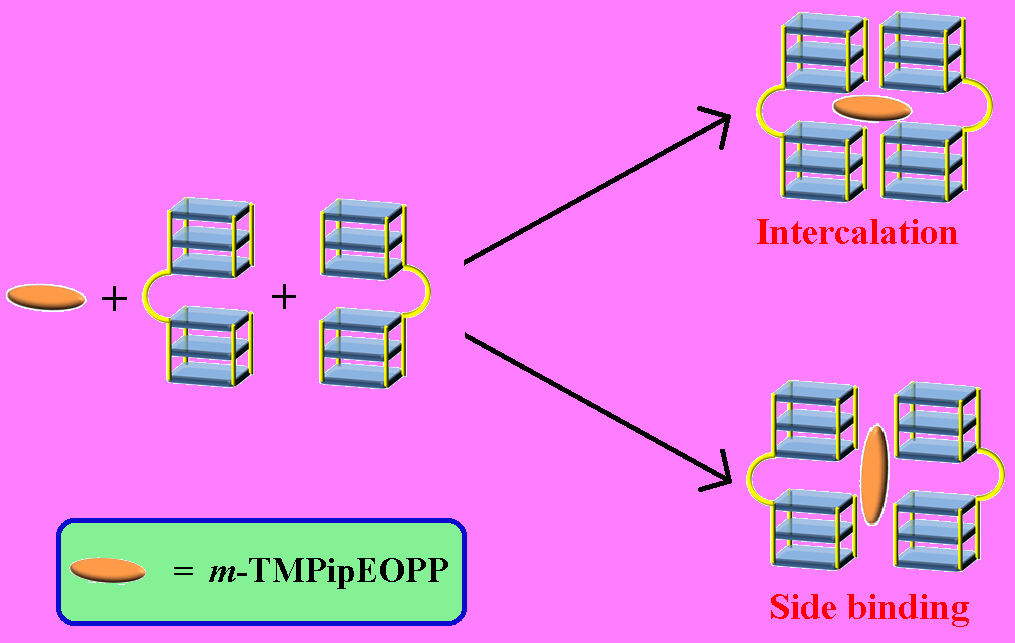


Scheme S1. Two possible binding modes between *m*-TMPipEOPP and multimeric G-quadruplexes.

1. G-quadruplex-stabilizing abilities of ***m*-TMPipEOPP to G-quadruplexes**

10.1 Under dilute conditions

**Figure S43.** Melting temperature (*T*_1/2_) detection of G-quadruplexes in the absence (black) and presence (red) of 5 μM *m*-TMPipEOPP under dilute conditions. Scatter: experimental data, line: fitting curves.

10.2 Under molecular crowding conditions

**Figure S44.** Melting temperature (*T*_1/2_) detection of G-quadruplexes in the absence (black) and presence (red) of 5 μM *m*-TMPipEOPP under molecular crowding conditions. Scatter: experimental data, line: fitting curves.

1. Stabilities of multimeric G-quadruplexes with different pocket sizes

**11.1 Under dilute conditions**

**Figure S45.** Melting temperature detection of multimeric G-quadruplexes with different pocket sizes under dilute conditions. Scatter: experimental data, line: fitting curves.

**11.2 Under molecular crowding conditions**

**Figure S46.** Melting temperature detection of multimeric G-quadruplexes with different pocket sizes under molecular crowding conditions. Scatter: experimental data, line: fitting curves.
